# New York State Veterinary Medical Society

**Published:** 1895-02

**Authors:** Nelson P. Hinkley

**Affiliations:** Secretary


### SOCIETY PROCEEDINGS.

### THE NEW YORK STATE VETERINARY MEDICAL SOCIETY.

FIRST DAY. *Wednesday, September 12, 1894.*

The fifth annual convention was held at the Delavan House, Albany, September 12 and 13, 1894. The meeting was called to order by the President, Dr. R. S. Huidekoper, at 2 P. M.

On roll-call by the Secretary the following members were present: Drs. John A. Bell, W. L. Baker, James Carnrite, Charles Cowie, W. H. Carpenter, N. P. Hinkley, E. D. Hayden, R. S. Huidekoper, E. B. Ingalls, Claude D. Morris, William Machan, John Wende, R. D. Austen, F. Morrow, W. J. Wadsworth, V. L. James, E. J. McLeod, Thomas Giffin, J. W. Darby, William H. Kelly.

The President then addressed the meeting, calling the attention of the members present to the growing importance of veterinary organization, and urging all members to form local societies and try to induce members of the profession to become members of county, State, and United States veterinary societies.

He called attention to the valuable information gained and the many advantages to be derived from such meetings, not only from debates and discussions on papers and communications read before the meeting, but also by the free exchange of ideas and individual thoughts. He said it also gives us all an opportunity to become better acquainted with our fellow practitioners both socially and professionally, and he always felt, when arriving home after attending some veterinary society meeting, as though his time had been well and usefully spent. He hoped that this meeting would be one of interest to every member present, and that the reports from the several committees, and the reading of the different papers, would bring forth from every member in the room discussions and knowledge sufficient to amply repay every one for attending. He briefly outlined the rapid and successful advance made by the veterinary profession in America; the reorganization of short-term schools, by demanding of its students a moderately strict matriculation examination, also lengthen their term of instruction from two sessions of from four to six months each to three sessions of six months each. He concluded by stating the great need of higher education, perfection, integrity, and ethics in the veterinary profession.

At the conclusion of the President's remarks the minutes of the last meeting were read by the Secretary.

Dr. Wende objected to certain parts of the minutes which quoted Dr. Wende as saying that a man from Ohio informed him that the staff of the Ohio Veterinary College was composed of gypsy horse dealers and blacksmiths. Dr. Wende said that what he did say was that he was informed by a man from Ohio that stockholders of the Ohio Veterinary College (not the staff) was composed of horse dealers, gypsies, and blacksmiths, and asked the Secretary to make these corrections. With these correction, it was moved by Dr. Bell, and seconded by Dr. James, that the minutes be accepted. Carried.

A recess of twenty minutes was then taken to consider the application of new members.

The following gentlemen's names were handed the Board of Censors to act upon: Dr. Arthur O'Shea, New York City; vouched for by Drs. Thomas Giffin and William Machan. Dr. H. B. Ambler, Chatham, New York; vouched for by Drs. Claude D. Morris and John Wende. Dr. J. H. Huhne, Kingston, New York; vouched for by Drs. R. S. Huidekoper and N. P. Hinkley. Dr. W. W. Kennedy, Fulton, New York; vouched for by Drs. M. M. Poucher and James A. Pendergrast. Dr. Arthur W. Baker, Cortland, New York; vouched for by Drs. Claude D. Morris and John Wende. Dr. A. G. Wicks, Schnenectady, New York; vouched for by Drs. Claude D. Morris and W. L. Baker. Dr. W. B. Switzer, Willows, New York; vouched for by Drs. D. C. Papworth and E. H. Nodyne. Dr. J. M. Currie, Rome, New York; vouched for by Drs. Frank Morrow and John Wende.

The Board of Censors went into executive session, and at 4.30 P.M. the President called the meeting to order, and the chairman of the Board of Censors, Dr. Claude D. Morris, reported favorably upon the following names: Drs. Arthur O'Shea, H. B. Ambler, J. H. Huhne, W. W. Kennedy, Arthur W. Baker, A. G. Wicks, W. B. Switzer, J. M. Currie.

It was moved by Dr. Morris, and seconded by Dr. Morrow, that the names here reported by the Board of Censors be balloted for. Carried. The Chair then appointed Drs. Morrow and Machan as tellers. A separate ballot was taken upon each individual name and, no dissenting ballot being cast, they were all elected members. The new members were then brought into the assembly room and introduced by the President.

### Reports of Committees.

The Committee on Legislation, Dr. W. L. Baker, reported that the committee had met at Albany and had done good work, although they had not accomplished their aim and object, nevertheless, they had succeeded in making their wants better known to the several members of the legislative body, and had materially increased the number of our friends and co-workers in our cause. Dr. Baker, in his report, forcibly urged all veterinarians in New York State who are in sympathy with our cause and desire to elevate the profession and to aid in abolishing quackery, illegal practice, and illiterate charlatans, to come forward and put their shoulders to the wheel and give the Committee on Legislation every assistance, not only by their personal aid and influence but to interest all of their friends and members of the Assembly and Senate in our cause. Explain to them our wants and our desires, and you can rest assured that few men who are intelligently informed that we are asking of them to pass a law for the public and our protection but will be with us when the time comes for the casting of their votes. Dr. Baker continued by asking for an extension of time for the Committee on Legislation, and finished by calling the attention of the members present that, seeing that Prof. Law was unavoidably absent, he, Dr. Baker, wanted to say that the society was indebted to Prof. Law for his personal endeavors during last winter in trying to get our proposed bill passed, and that the Professor had worked with untiring energy to see the bill become a law, and Dr. Baker was sure that the Professor would again willingly take the same interest the coming year.

Dr. Baker's report was received with applause. It was moved by Dr. Morris that the report be accepted and the committee be given more time for work. This motion was seconded by Dr. Morrow and was carried.

Dr. Arthur O'Shea, of New York, made a few important and interesting remarks on his experience in Albany in his efforts to improve the present veterinary laws as they now stand. He stated that after being in Albany a short time he discovered the very unpleasant fact that the empirics had nearly succeeded in having the old law of registration extended to January. 1895. After enlisting the aid of several State officials in his cause he had the gratifying assurance from the principal members of the Legislature that the time for registration would not be extended.

Dr. O'Shea continued his remarks by urging all veterinarians in New York State to arouse themselves and put new energy in our cause, see their representative members of Assembly, explain the condition of the proposed law pertaining to the regulation of veterinary practice in this State, also the reasons why we, as a veterinary body, ask for better laws for our professional protection and the better protection of the health of the people of the entire State. Dr. O'Shea, in concluding his remarks, spoke of another beneficial change (which would benefit the veterinarian) in the laws exempting the veterinarian from jury duty. This was simply an amendment of a law which had formerly exempted medical and some other professional men from acting as jurors. The Doctor said before closing his remarks that he would like to urge the Committee on Legislation not to wait until the eleventh hour of the session at Albany, but to be on the field early and come prepared to stay until they had either lost or succeeded in gaining their cause.

Dr. Claude D. Morris said, inasmuch as Dr. O'Shea had saved the veterinarians of the State of New York the disgrace of having the disgraceful law of 1886 extended, that he took pleasure in offering the following resolutions:

*Resolved*, That this society desire to extend to Dr. O'Shea, of New York City, their hearty approval and congratulations in testimony of his valuable services in behalf of elevating our honored profession.

*Resolved*, That by his timely interest and energy he succeeded in preventing further advances made on the part of and in behalf of empirics of our State to gain both illegal and unwarranted recognition in the profession under the guise of statutory provisions.

*Resolved*, That we recognize these valuable services, and that we take pleasure, in convention assembled, to so express our approval.

These resolutions were seconded by Dr. Baker and unanimously carried. Dr. O'Shea then expressed his appreciation of the same.

On motion of Dr. Baker, seconded, the meeting adjourned at 1.10 P.M. to come together again at 2.30 P.M.

*Afternoon Session.*

The meeting was called to order at 2 P.M. with a full attendance of all members. The next business before the meeting was the report of the Committee on Publication. The chairman, Dr. Hinkley, reported progress, and said that he expected soon to be able to send to each member a copy of the revised by-laws and constitution, as the committee desired to add a short history of the organization, a list of members, and considerable other useful information in the same pamphlet. He therefore would ask for an extension of time. Dr. Wende moved that the report be accepted and the committee be given more time; seconded by Dr. Cowie. Carried.

The Committee on Prosecution, Dr. Wende, chairman, reported some cases in Erie and neighboring counties, where illegal practising had been reported to the committee. He also said that one or two test cases were about to be tried. He pointed out some difficulties of always obtaining sufficient evidence of the proper kind to convict a defendant under the present law. He concluded by saying that the committee would have more and important information at the next meeting of the society.

A very interesting discussion followed the report of this committee, which was taken part in by every member in the room. It was evident from the remarks made by the several members that quackery and illegal practising were very prevalent throughout the State, and that the proposed bill, of which our Society has been so anxious and have made such earnest attempts to have become a law, is one of the necessary and important questions for the Committee on Legislation to urge and work for.

Moved by Dr. James, and seconded by Dr. Carnrite, that the report of this committee be accepted, and the members be retained and asked to continue on with their good work and report at our next meeting.

*Report of Committee on Arrangements.* Dr. Kelly, chairman of this committee, reported what the committee had done to arrange for the present meeting, and informed the members present of several invitations from State officers to visit the Capitol and other places of interest in the city.

Moved by Dr. Darby, and seconded by Dr. Hayden, that the report of this committee be accepted, and also the invitation to visit the different places of interest, and that the committee be tendered a vote of thanks for the excellent arrangements made by them. Carried.

*Report of County Secretaries.* The Secretaries of the several County Societies reported excellent progress, many of them forming strong county societies, having regular meetings with good attendance, creating a much more friendly feeling in the profession, and also reported that the Public Boards of Health and the public in general are all taking an active interest in the welfare of our profession, and acknowledge the vast good that we are doing for the prevention of the spread of contagious diseases of our domestic animals, and the important part that the profession is taking in preventing and stamping out diseases of animals that affect the human health by way of communicating the disease through the consumption of affected, diseased meats and milk, and the great danger arising from such a condition of our public dairies and abattoirs. It shows that the public are rapidly becoming educated and aware that for their own protection and their good health it is necessary that qualified and competent veterinarians be appointed to places of trust and importance on all our health boards, cattle and dairy commissions, and in fact on all boards and committees whose duties are to investigate and regulate the public health through food and food-products, and that the veterinarian be more often consulted by the Public Health Board and private owners of animals in relation to contagious diseases, etc.; that it is evident that the great factor of the coming veterinarian would be to prevent disease rather than to cure. Several of the secretaries of the different county societies reported fully on the recent investigation of tuberculosis by State authorities in this State. This brought out a long and interesting discussion on the manner of investigation, the great good it was doing, and the future need of a general investigation being taken charge of by the National Government under the jurisdiction of the Bureau of Animal Industry, the systematic inspection, examination, and testing by tuberculin of every herd of cattle in the State of New York, certificates being issued to the owners certifying to those proven free from tuberculosis. Those showing by careful testing that they are affected with tuberculosis to be promptly condemned, and after an official autopsy being held, the carcass to be cremated. Also to have a law passed compelling all cattle entering into the State of New York from other States, Territories, or foreign countries, to be accompanied with a certificate from the State Board of Health, or other duly authorized bodies, certifying that the said animals had been recently tested with tuberculin and were found free from tuberculosis. Also to have all cattle which are used for milk-producing purposes, or the milk of which was used for public consumption, regularly examined by a competent veterinarian with the tuberculin test. In this way the people of the State of New York would be positive that every quart of milk offered for sale or consumed in the Empire State would be free from tubercle bacilli.

It was moved by Dr. Baker and seconded by Dr. Morrow, that the reports of the several county secretaries be accepted and that they continue as secretaries of their respective counties, and report at the next meeting. Carried unanimously.

*Miscellaneous business.* Under this head the question arose as to delinquent members who were back in their dues to the Society and who showed no inclination either to pay or to attend the meetings. This was brought before the members for their consideration and action by the Secretary, Dr. Hinkley. After quite a discussion by all the members present, the President, Dr. Huidekoper suggested that delinquent members be dealt with according to the by-laws governing this matter.

It was moved by Dr. Wende and seconded by Dr. Cowie, that the Secretary be instructed to write all members in arrears for all dues, assessments, and initiation fees, calling their attention to the by-laws governing this matter, and to furnish a list of all members who were still in arrears, and that the Society then and there suspend them. Carried unanimously.

The Secretary then read several letters and telegrams of regret from several members who were unavoidably absent and who were unable to attend this meeting. They were from Prof. James Law, Dr. Nodyne, Dr. Burns, and others.

Dr. E. J. McLeod moved, and it was seconded by Dr. Wende, that the meeting adjourn until 10 A.M., September 13th; carried.

SECOND DAY, *Thursday, September* 13, 1894.

The meeting was called to order at 10 A.M., President Dr. R. S. Huidekoper in the chair. The following gentlemen answered to their respective names: Drs. John A. Bell, W. L. Baker, James Carnrite, Charles Cowie, W. H. Carpenter, N. P. Hinkley, E. D. Hayden, R. S. Huidekoper, E. B. Ingalls, Claude D. Morris, William Machan, John Wende, R. D. Austin, F. Morrow, W. J. Wadsworth, V. L. James, E. J. McLeod, Thomas Giffin, J.

W. Darby, William H. Kelly, J. M. Currie, A. G. Wicks, Arthur O'Shea, J. H. Huhne.

The minutes of the meeting of the previous day were read by the Secretary and a motion was made by Dr. Morrow, seconded by Dr. James, that they be accepted; carried.

It was moved by Dr. Morris, and seconded by Dr. Baker, that, inasmuch as the reading of the papers was the next event in the order of business, that all the papers be read by their authors before any discussion, and after the reading of all the papers the discussion of the papers be taken in turn as they were read; carried.

The President now called on Dr. Claude D. Morris to read his paper on "Meat Inspection."[1]

At the conclusion of the reading of Dr. Morris's paper, which was listened to with very great interest, and was received with applause, the President asked for the reading of Professor Liautard's paper, "Veterinary Education in Relation to the Practice of Veterinary Medicine in New York State." On account of the unavoidable absence of Professor Liautard, the Secretary, Dr. N. P. Hinkley, was requested by the President to read the Professor's paper.

At the conclusion of the reading of Professor Liautard's paper, which was well received, as was shown by the frequent applause as the Secretary read the paper, and on account of the absence of the other essayists, Dr. Berns, Dr. Nodyne, Dr. Chase, and Prof. James Law, and of the very great importance of the subject of the two papers before the meeting now for discussion, it was suggested by the President that the discussion take place in the afternoon session.

It was therefore moved by Dr. Carnrite, and seconded by Dr. Austen, that the meeting adjourn until 2 P.M.

*Afternoon Session.*

The meeting was called to order by the President, Dr. R. S. Huidekoper, at 2 P.M. sharp, with a full attendance of all the members who were present at the morning session. The President remarked that the discussion of Dr. Claude D. Morris' paper on "Meat Inspection" was now in order. A most animated and interesting discussion followed, which was taken part in by every member in the room, and it was evident by the discussion that followed that the subject that Dr. Morris had chosen was one that had been interesting to all the members of our profession. It showed that Dr. Morris had lost none of his past ability to ably write and defend a paper before a body of veterinarians, which not only proved interesting, but also highly instructive.

The discussion of Professor Liautard's paper was next in order, and was so announced by the President, and almost immediately several of the members present were on their feet at once, asking for recognition from the President. It was evident that the Professor had written on a subject that was not only interesting to his listeners, but was a matter of great importance to the future of the veterinary profession in this State. It was apparent by the long and lively discussion that followed that the Professor's excellent paper,

[1] Printed in this number.

in which he stated so many facts in regard to the past history of veterinary education in the State of New York, and the laborious work and the many difficulties and the enormous responsibility which rested on the shoulders of the organizers of the veterinary schools in this State of over a quarter of a century ago, showed the Professor not only to be a pioneer of veterinary education, but also, from his past history and records, showed him to be one of the most earnest, honest, and energetic workers in behalf and for the good, promotion, and elevation of the veterinary profession in this State. The Professor's paper found many supporters among the members who took part in the discussion, and argued that it was well to go slow and careful with the proposed veterinary legislation, and not to enact any law that would injure and retard the good work in veterinary education that is being done yearly by some of our recognized veterinary schools in this State.

Dr. Cowie said that it would give him much pleasure to make a motion that Professor Liautard and Dr. Claude D. Morris be given a vote of thanks for their two very excellent papers, both of whose subjects were of so much importance, not alone to the veterinary profession, but to the public at large in this State.

This was seconded by Dr. Baker and carried unanimously.

*Unfinished business.* Under this head Dr. Baker offered the following resolution: Inasmuch as certain members of our Society have notified and promised the Secretary of this Society that they would write, read, and defend certain essays on their chosen subjects at this annual meeting to be held at Albany, N. Y., September 12 and 13, 1894, the failure of said members to either attend, send papers, or excuses to the Secretary, stating why they could not attend the meeting, and by such flagrant neglect have caused an annoyance to the attending members of the Society; be it

*Resolved*, That members having essays to read be earnestly requested either to be present to read and defend their papers or send said papers or a written notice of their inability to do so, and not subject the members of the Society to the above-mentioned annoyance and delay.

Dr. Baker's resolution was unanimously adopted. Moved by Dr. Morrow and seconded by Dr. Baker that the proprietors of the Delavan House be given a vote of thanks for the courteous and kind treatment to the members of the Society, and for the gratuitous donation of their assembly parlors to the Society This was carried unanimously and the Secretary directed to convey the expressions of the Society to the proprietors.

*Place of meeting.* This being the next order of business, after a very spirited discussion, in which several of the members proposed Rochester, Syracuse, Buffalo, Troy, Elmira, and New York City, it was placed before the Society in the form of a vote, and the members in favor of New York City were in the majority. The result was 20 for New York City and 2 for Syracuse.

The President appointed Drs. Hinkley, Wende, Baker, and Machan as delegates to the United States Veterinary Society meeting. As there was no more business before the meeting, Dr. James made a motion, which was seconded by Dr. Hayden, that the meeting adjourn to meet again in New York City in the month of September, 1895. Date and place of meeting were left to the discretion of the Secretary and President in conformity to the by-laws governing this matter. Adjourned.

NELSON P. HINKLEY,
Secretary.

